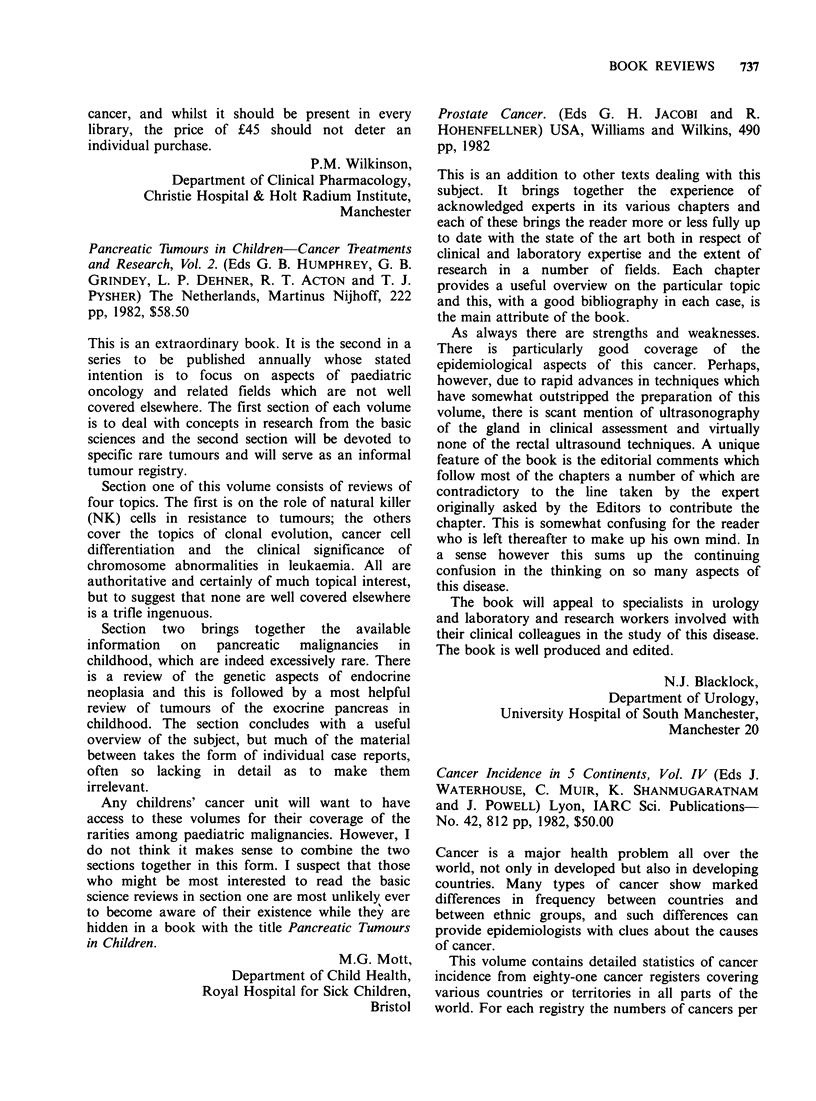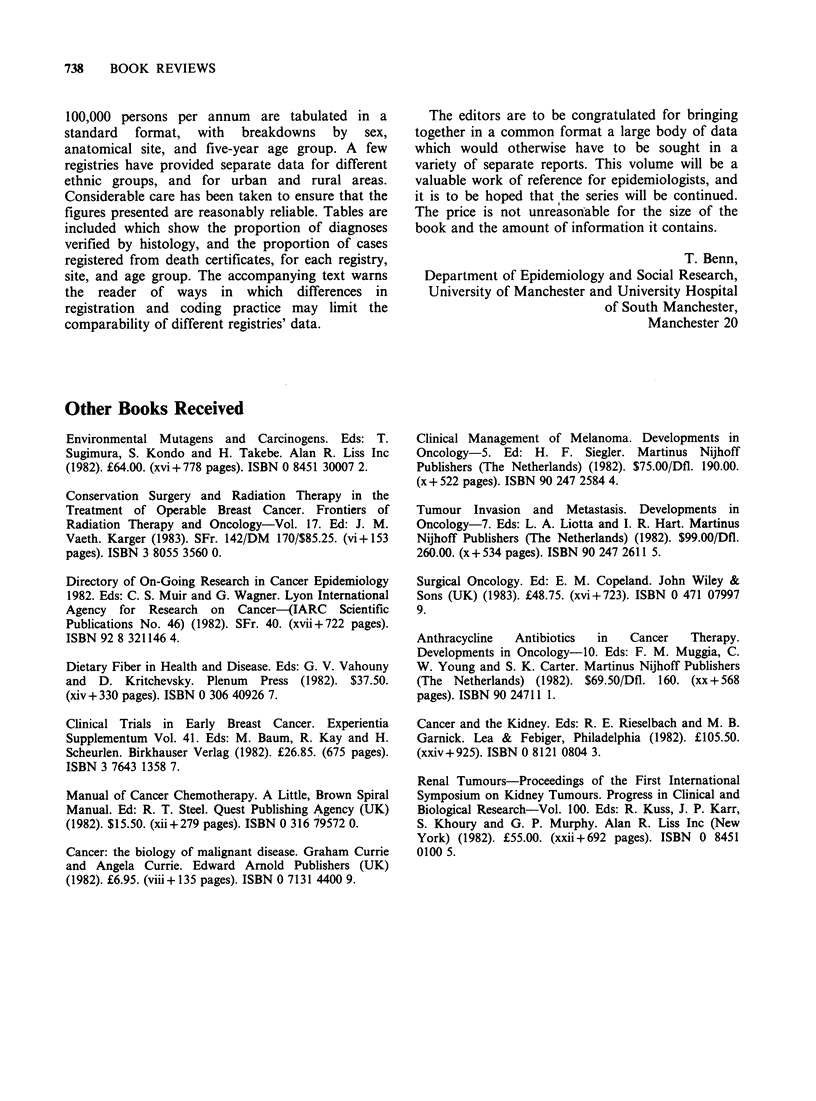# Cancer Incidence in 5 Continents, Vol. IV

**Published:** 1983-05

**Authors:** 


University Hospital of South Manchester,

Manchester 20

*Cancer Incidence in 5 Continents, Vol. IV* (Eds J. WATERHOUSE, C. MUIR, K. SHANMUGARATNAM and J. POWELL) Lyon, IARC Sci. Publications—No. 42, 812 pp, 1982, $50.00

Cancer is a major health problem all over the world, not only in developed but also in developing countries. Many types of cancer show marked differences in frequency between countries and between ethnic groups, and such differences can provide epidemiologists with clues about the causes of cancer.

This volume contains detailed statistics of cancer incidence from eighty-one cancer registers covering various countries or territories in all parts of the world. For each registry the numbers of cancers per

100,000 persons per annum are tabulated in a standard format, with breakdowns by sex, anatomical site, and five-year age group. A few registries have provided separate data for different ethnic groups, and for urban and rural areas. Considerable care has been taken to ensure that the figures presented are reasonably reliable. Tables are included which show the proportion of diagnoses verified by histology, and the proportion of cases registered from death certificates, for each registry, site, and age group. The accompanying text warns the reader of ways in which differences in registration and coding practice may limit the comparability of different registries' data.

The editors are to be congratulated for bringing together in a common format a large body of data which would otherwise have to be sought in a variety of separate reports. This volume will be a valuable work of reference for epidemiologists, and it is to be hoped that the series will be continued. The price is not unreasonable for the size of the book and the amount of information it contains.

T. Benn,
Department of Epidemiology and Social Research,
University of Manchester and University Hospital
of South Manchester,
Manchester 20